# Resistant Starch Combined with Whey Protein Increases Postprandial Metabolism and Lowers Glucose and Insulin Responses in Healthy Adult Men

**DOI:** 10.3390/foods10030537

**Published:** 2021-03-05

**Authors:** Alex E. Mohr, Olivia Minicucci, Dale Long, Vincent J. Miller, Allison Keller, Caitlin Sheridan, Gabriel O’brien, Emery Ward, Brad Schuler, Scott Connelly, Jens J. Holst, Arne Astrup, Feng He, Christopher L. Gentile, Paul J. Arciero

**Affiliations:** 1 Human Nutrition and Metabolism Laboratory, Department of Health and Human Physiological Sciences, Skidmore College, Saratoga Springs, NY 12866, USA; aemohr@asu.edu (A.E.M.); ominicucci1@gmail.com (O.M.); dalelong@gmail.com (D.L.J.); vin.miller@gmail.com (V.J.M.); allie.keller12@gmail.com (A.K.); caty.sheridan@gmail.com (C.S.); gobrien@skidmore.edu (G.O.); emeryward@gmail.com (E.W.); schulerbrad@gmail.com (B.S.); fhe@csuchico.edu (F.H.); 2 College of Health Solutions, Arizona State University, Phoenix, AZ 85004, USA; 3 Scott Connelly Foundation, Corona Del Mar, Newport Beach, CA 92625, USA; ascottcmd@gmail.com; 4 Department of Biomedical Sciences, University of Copenhagen, 1017 Copenhagen, Denmark; jjholst@sund.ku.dk; 5 Department of Nutrition, Exercise and Sports, University of Copenhagen, 1017 Copenhagen, Denmark; ast@nexs.ku.dk; 6 Department of Kinesiology, California State University, Chico, CA 95929, USA; 7 Department of Food Science and Human Nutrition, Colorado State University, Fort Collins, CO 80523, USA; christopher.gentile@colostate.edu

**Keywords:** thermic effect of food, fuel utilization, resistant starch, whey protein, energy expenditure, hunger

## Abstract

Resistant starch (RS) and/or protein consumption favorably influence energy metabolism, substrate utilization, and weight management. The current study administered four different versions of a pancake breakfast containing waxy maize or RS with and without whey protein (WP) and measured postprandial thermogenesis (TEM), fuel utilization, and circulating satiation and appetite factors for 180 min in a group of healthy, adult men. On four separate visits to the laboratory, eight participants were administered four different pancake breakfast meal challenges using a single-blind, randomized crossover design: (1) waxy maize starch (WMS) control; (2) WMS and WP (WMS + WP); (3) RS; or (4) RS and WP (RS + WP). TEM (kcals/180 min) was significantly greater (*p* < 0.05) in RS + WP (45.11; confidence interval (CI), 33.81–56.41) compared to WMS (25.61; CI, 14.31–36.91), RS (29.44; CI, 18.14–40.74), and WMS + WP (24.64; CI, 13.34–35.94), respectively. Fat oxidation was enhanced (*p* < 0.05) after RS + WP compared to RS at 60 min (+23.10%), WMS at 120 min (+27.49%), and WMS and WMS + WP at 180 min (+35.76%; +17.31%, respectively), and RER was decreased with RS + WP versus the other three meals (mean differences: ≥−0.021). Insulin concentrations were decreased (*p* < 0.05) following RS + WP compared to WMS, whereas both RS (−46.19%) and RS + WP (−53.05%) insulin area under the curve (AUC) were greatly reduced (*p* < 0.01) compared to WMS. While limited by sample size, meals containing both RS and WP increased postprandial thermogenesis and fat oxidation, and lowered insulin response compared to isocaloric meals without this combination. Therefore, RS + WP may favorably impact energy metabolism and thus weight control and body composition under chronic feeding conditions.

## 1. Introduction

The increasing obesity epidemic has prompted researchers to seek novel nutritional strategies to manage a healthy body weight and composition. While dietary approaches such as macronutrient composition, caloric restriction, and meal frequency have been popular areas of focus, the use of specific food products and dietary components are increasingly showing promise in the treatment of obesity [1,2]. Of these, dairy protein fractions, including whey protein (WP), and indigestible carbohydrates, like resistant starch (RS), are both increasingly being viewed as strategies to control body weight and enhance body composition when incorporated into a nutritional intervention [3,4,5,6].

As a category, RS is starch that moves through the small intestine to the colon undigested/unabsorbed, where it can be metabolized through microbial fermentation processes and ultimately produce short-chain fatty acids (SCFAs) as well as small microbes with health-promoting properties [7]. Multiple studies in animals [8,9,10,11], and a growing body of evidence in humans [12,13,14], including from our lab [6], report that dietary RS (obtained from RS4 hydroxypropyl distarch phosphate) may support healthy body weight management. There are several proposed mechanisms of action, including (1) the ability of RS to reduce the number of metabolizable calories because of its resistance to digestion and absorption [9]; (2) the production of SCFAs, which may increase the absolute number of calories burned and lipid oxidation [15]; and (3) reduction in calories consumed by promoting release of appetite and hunger signals, including gastro-entero-hepatic hormones peptide YY (PYY) and glucagon-like polypeptide-1 (GLP-1), both of which bind receptors in the brain and decrease appetite and serve as anorexigenics [16]. In addition, clinical research has reported reductions in fasting and postprandial glucose levels and enhanced insulin sensitivity and gut-derived satiety peptides [17].

There are four types of RS: RS found in seeds, germs, and whole grains that is resistant to digestive enzymes (RS1); high-amylose starch from maize that contains α-1,4 glycosidic bonds (RS2); starch from cooked and cooled grains such as pasta and rice that has been retrograded (RS3); and starch that has been chemically modified (RS4, hereinafter RS; [18]). Some of these forms can be found naturally in the human diet, including RS1, provided by whole grains (rice, pasta). RS2 is derived from raw potatoes and unripe bananas and resists carbohydrate digestive enzymes. RS3 is retrograded starch that occurs after grains, such as rice and pasta, have been cooked then cooled [3]. In contrast, RS4 is starch that is chemically altered and more resistant to retrogradation, with increased viscosity, but it is more stable in response to acid and increased temperature compared to naturally occurring starch [3]. While less studied than other forms, RS4 consumption increases resting energy expenditure and lipid utilization in lean men [19] and improves post-meal glucose and insulin levels in healthy adults [20]. Therefore, RS4 may offer an attractive candidate as a potential functional ingredient in various food applications replacing other forms of starch with lower overall nutritional value.

Increasing the intake of dietary protein over the current recommendations is another nutrition-focused strategy proposed for weight maintenance and optimal body composition [21,22]. As with RS, increased dietary protein is associated with increased resting metabolism and satiety [23,24]. Previously, our lab showed higher quantity and frequency of dietary protein (termed “protein pacing”) optimizes fat loss and muscle mass retention along with elevating caloric expenditure in obese adults [4]. Thus, combining RS, obtained from RS4 type hydroxypropyl distarch phosphate, and dietary protein, specifically WP, may offer a supra-additive thermogenic and metabolic advantage beyond each functional food alone. Indeed, our previous study was the first, and only one, to demonstrate an acute meal challenge combining dietary RS and WP increases fat oxidation, PYY, satiety and fullness compared to waxy maize starch control (WMS), WMS + WP, and RS alone in women [6]. While the results of this research were notable, males were not assessed. Therefore, the current study examined breakfast meal challenges containing waxy maize (non-RS) ingredients or RS with and without added WP on fuel utilization, postprandial thermogenesis, subjective measures of hunger and appetite, and circulating signals of satiety hormones in healthy men. For this purpose, and similar to our previous study, we conducted a randomized, single-blind, crossover design with four meal challenges consisting of pancake meals provided for breakfast with (1) WMS (control); (2) WMS + WP; (3) RS; or (4) RS + WP.

## 2. Materials and Methods

### 2.1. Participants

A total of 70 men were recruited through newspaper advertisements and flyers from the Saratoga Springs, NY area and initially screened for enrollment. After screening, 24 were eligible for participation. Due to scheduling and time constraints, as well as funding limitations, 8 men completed all four test meal challenges. Participants were generally healthy, non-smokers, with no obvious cardiometabolic disease per medical history and personal physician clearance. In addition, participants were weight stable (±2 kg) > 6 months before the study started and provided informed written consent in accordance with Skidmore College Human Subjects Institutional Review Board before participation. The experimental procedures adhered with Federal Wide Assurance and related New York State regulations, that are in line with the National Commission for the Protection of Human Subjects of Biomedical and Behavioral Research and in agreement with the Helsinki Declaration as revised in 1983. This trial was registered at clinicaltrials.gov as NCT02418429.

### 2.2. Pancake Test Meal

Participants consumed one of four pancake test meals at the Human Nutrition and Metabolism Laboratory in a single-blind, randomized repeated measures crossover design, separated by at least four days between meals. The four pancake test meals included (1) waxy maize (control) starch (WMS, *n* = 8); (2) waxy maize starch and whey protein (WMS + WP, *n* = 8); (3) resistant starch (RS, *n* = 8); or (4) RS and whey protein (RS + WP, *n* = 8). The whey protein concentrate (Hilmar 8610 high gel whey protein concentrate; Hilmar CA) was derived from dairy whey processed by a cross-flow filtration process resulting in a leucine content of 8.5 g per 100 g. All pancake test meals were consumed together with water (180 mL) only and prepared according to institutional guidelines. All ingredients were combined and mixed together to create a batter that consisted of gelatinized test starch, sugar, maltodextrin, vegetable oil, baking powder, egg, non-fat dry milk powder, and water. Participants were instructed to consume three pancakes cooked on a non-stick griddle. Table 1 shows the specific nutritional profile of the four pancake breakfast test meals.

### 2.3. Experimental Design

**Figure 1:**
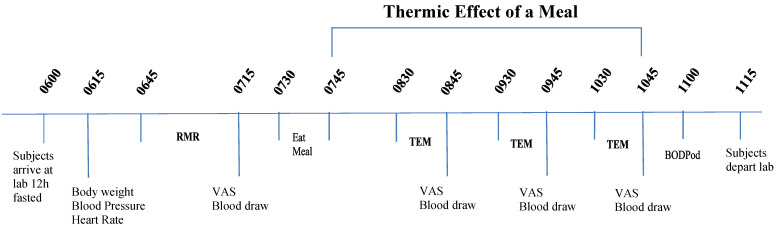
Timeline of pancake test meals for thermic effect of a meal (TEM). RMR, resting metabolic rate; VAS, visual analog scales for hunger, fullness, satiation, desire to eat.

One day prior to each of the four pancake breakfast test meal visits, all participants prepared their own meals at home by adhering to a menu plan outlined by a nutritionist and based on the participant’s estimated caloric needs (~25% protein, ~50% carbohydrate, and ~25% fat). The dinner meal the day before each of the four breakfast test meals was identical and consumed between 1800 and 2000 h. All laboratory testing initiated between 0600 and 0700 following a 12 h fast (water only) and at least 24 h removed from strenuous physical activity, caffeine, and alcohol consumption (Figure 1).

The morning of the test meals, participants arrived to the laboratory, and body weight was recorded (Befour Inc., Saukville, WI, USA, model number FS0900), followed by 15 min of quiet supine resting in a dimly lit room. Resting metabolic rate (RMR) was then measured for 30 min. A fasted blood draw was obtained for measures of plasma insulin, glucose, gastric inhibitory peptide (GIP), glucagon like polypeptide-1 (GLP-1), peptide YY (PYY), and ghrelin (GRL), and subjects completed visual analog scales (VAS) for ratings of hunger, desire to eat, and satiety (see testing procedures below). One of the four test meals was then consumed (1: WMS; 2: WMS + WP; 3: RS; 4: RS + WP) within 12 min followed by supine resting for 180 min (3 h). Blood samples were collected, VAS were completed (minutes 60, 120, 180), and the thermic effect of the meal (TEM) (45–60, 105–120, 165–180 min) via indirect calorimetry was obtained as reported previously by Arciero et al. [4]. After the first test meal was completed, body composition was measured (Life Measurements BODPod Body Composition Tracking System, Concord, CA, USA).

### 2.4. Resting Metabolic Rate (RMR) and Thermic Effect of a Meal (TEM)

RMR was measured with a computerized open-circuit indirect calorimeter (Parvomedics, Truemax 2400, Salt Lake City, UT, USA) as previously described [6]. Measurements were captured with participants supine, but not allowed to sleep, in a thermo-neutral, dimly lit room. TEM included postprandial thermogenesis every 45 min for 180 min (TEM 45–60; 105–120; 165–180 min). The last 10 min of each 15 min block was averaged for the TEM, and the total 180 min TEM was derived by averaging each 10 min TEM measurement and multiplying by 60 min (0–60; 61–120; 121–180 min). Each three 60 min TEM period was then summated to calculate the 180 min TEM value. Test-retest intraclass correlation (r) and coefficient of variation (CV) for RMR (Kcal/min) was *R* = 0.92 and 4.2%, respectively.

The respiratory exchange ratio (RER) and fuel utilization were similarly derived using indirect calorimetry data. This provided fuel oxidation rates (lipid and carbohydrate) using standardized caloric equivalents. Total fat and carbohydrate oxidation rates were calculated using methodology described above for TEM. We chose a 180 min postprandial thermogenic measurement period (TEM) to capture the majority of the thermic response. Importantly, total calories consumed at the 4 pancake test meals were equal in kilocalories and different only in starch and protein composition (Table 1). This allowed for a direct comparison among all four pancake test meal conditions.

### 2.5. Plasma Biomarkers

Approximately 20 mL of venous blood was collected every hour from each participant during the three hour postprandial period (TEM, minutes 60, 120, 180). However, participant and resource constraints prevented sample collection for WMS + WP. Plasma samples were stored per standard procedure, as previously described [6]. All analytes (insulin, GRL, PYY, and GIP) were assessed with standard ELISA kits (Millipore, Inc. Burlington, MA, USA and DSL, Inc. Alpharetta, GA, USA), whereas plasma glucose concentrations were analyzed via the glucose oxidase technique (GM7 Analyser, Analox Instruments, Lunenberg, MA, USA). GLP-1 was determined with a radioimmunoassay treated with antiserum (no. 89390).

### 2.6. Feelings of Hunger, Satiation, and Desire to Eat

Subjective feelings of hunger, satiation, quantity of food that could be eaten, and desire to eat were all assessed with a visual analog scale (VAS) as previously described [6]. This technique uses a 100 mm line that is anchored at each end, and participants were instructed to place an “X” on the line indicating their levels of hunger, satiety, food quantity, and desire to eat. The distance of the “X” from the 0 mm point signified the level of sensation experienced at that time for each hunger rating. As an example, using hunger, an “X” at 0 mm indicated no hunger, while an “X” at 100 mm indicated extreme hunger. For all of the test meals, participants were instructed to complete VAS measures prior to the RMR and at 60 min intervals during the TEM meal tests (i.e., 60, 120, and 180 min).

### 2.7. Heart Rate and Blood Pressure

Participant’s resting heart rate and blood pressure were obtained by a trained investigator manually in the supine position after the RMR measurement on each of the four test days, as reported previously [25].

### 2.8. Statistical Analyses

To test normality assumptions, requisite statistics (Shapiro–Wilk tests and skewness and kurtosis z-scores) and probability plots (Q-Q plots and histograms) were generated. Where appropriate, log transformations were performed. Blood glucose, plasma insulin, and thermic effect of meal (TEM) area under curves (AUC) were calculated by the trapezoidal rule for the entire 3 h postprandial period [6,26], and absolute changes were determined by averaging baseline values (3 or 4 test meals) and subtracting from each post-meal value. Percent changes were calculated as the difference between baseline and each postprandial time point divided by the baseline value. The effect of each test meal on outcome variables used a linear mixed-effect model, with a random intercept for participant, time and meal type as fixed factors, and an interaction term (meal × time). Where appropriate, covariates were introduced to adjust for confounding. Based on marginal means for main (time and meal) and interaction effects (time × meal), multiple comparisons were made with Bonferroni post-hoc tests to protect against family-wise error and minimize the likelihood of significance due to multiple hypotheses. Power analysis and sample size was based on previous research [19]. Using an estimated effect size of *f* = 0.25, with power = 0.80 and α-level at 0.05, a sample size of 8 participants was determined to detect a significant time × meal effect in postprandial energy expenditure for a crossover design (G*Power 3.1). All analyses were performed using SPSS 26.0 for Windows (SPSS Inc., Chicago, IL, USA). An α-level was set at a significance of *p* < 0.05. Data are shown as mean values (with 95% confidence intervals (Cis)) unless otherwise noted.

## 3. Results

### 3.1. Participants and Compliance

Participants were middle-aged (51.4 ± 11.5 years), normal- and over-weight (BMI = 29.84 ± 7.77 kg/m^2^; percent body fat = 26.42 ± 11.62%). Baseline physical characteristics are shown in Table 2.

### 3.2. Assessment of Energy Intake

Participants maintained similar nutritional intakes the day before each laboratory test meal (data not shown). The macronutrient composition consisted of carbohydrates (50%), protein (25%), and fat (25%) at a level of intake that did not include the energy cost of physical activity. The reason for this was participants were instructed to refrain from physical activity the day prior to each laboratory testing day.

### 3.3. Resting Metabolic Rate, Thermic Effect of a Meal, and Substrate Utilization

**Figure 2:**
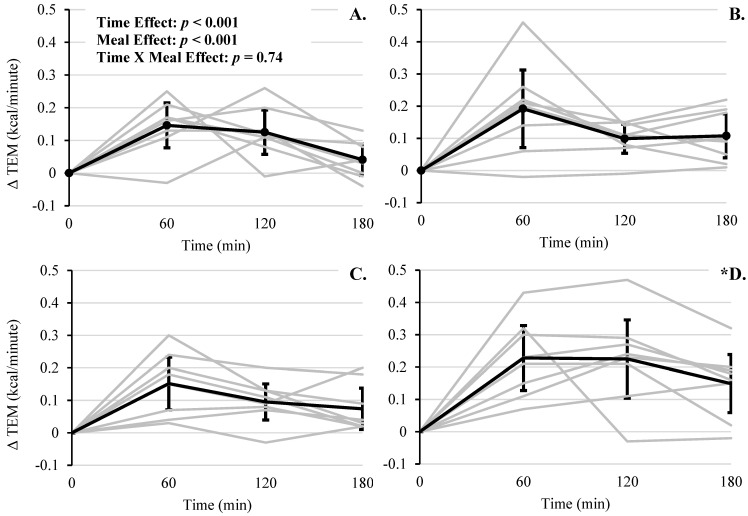
Postprandial change in TEM across 180 min for each meal. (**A**) WMS, (**B**) RS, (**C**) WMS + WP, and (**D**) RS + WP. Gray lines represent responses for each individual. Solid lines represent mean responses (95% upper and lower confidence interval). * Meal effects: RS + WP TEM > WMS, RS, and WMS + WP, *p* < 0.05.

There were significant main effects of time and meal type on TEM (kcal/minute) (*F*(3, 24) = 21.79, *p* < 0.001 and *F*(3, 96) = 11.27, *p* < 0.001, respectively; Figure 2).

Compared to RMR at baseline, postprandial TEM values were all significantly increased at 60 (+0.18 (95% CI, 0.11 to 0.25)), 120 (+0.14 (95% CI, 0.07 to 0.20)), and 180 min (+0.10 (95% CI, 0.03 to 0.16), all *p* ≤ 0.003). For meal type, TEM was significantly greater for RS + WP compared to the other conditions (mean differences: ≥0.07, all *p*’s ≤ 0.002; Table 3), with no other significant differences between meal types. Interestingly there was no significant interaction effect of meal × time (*F*(9, 96) = 0.67, *p* = 0.74).

**Figure 3:**
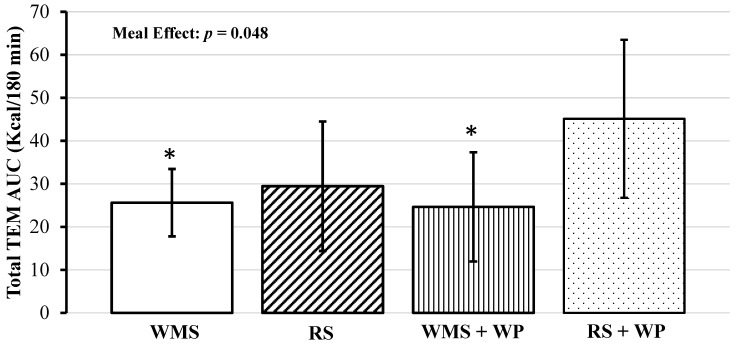
Comparison of thermic effect of meal (TEM, 180 min) area under the curve (AUC) for waxy maize starch (WMS), resistant starch (RS), waxy maize starch + whey protein (WMS + WP), and resistant starch + whey protein (RS + WP). * Significant difference compared to RS + WP *p* < 0.05. Data displayed as means ± 95% confidence intervals.

Notably, there were 4 TEM outliers, with z-scores ≥ 2.61. After removal, the interaction effect became slightly stronger, though remained nonsignificant (i.e., *p* = 0.17). Absolute AUC for TEM was significant (*F*(3, 32) = 2.94, *p* = 0.048), with RS + WP having a higher amount of calories burned compared to WMS (mean difference: +19.50 kcal/meal, (95% CI, 3.52 to 35.48), *p* = 0.018) and WMS + WP (mean difference: +20.48 kcal/meal, (95% CI, 4.49 to 36.46), *p* = 0.04) (Figure 3, Table 4), and there was a trend toward significance with RS + WP vs. RS (mean difference: +15.68 (95% CI, −0.30 to 31.66), *p* = 0.09).

**Figure 4:**
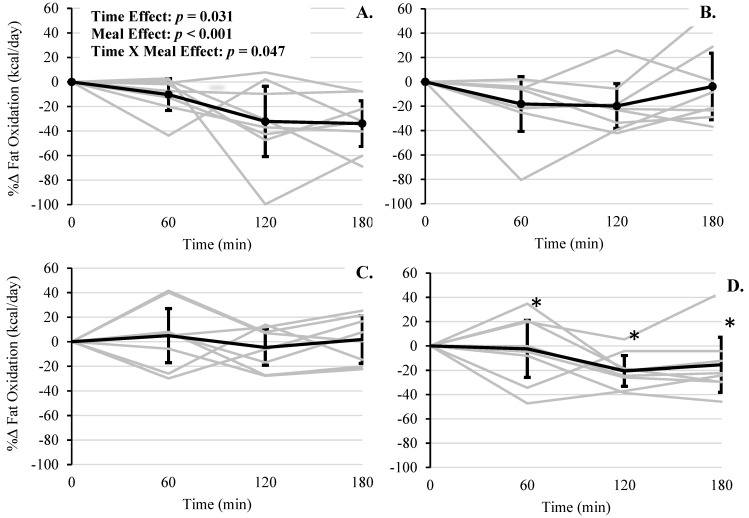
Postprandial change in percent fat oxidation across 180 min for each meal. (**A**) WMS, (**B**) RS, (**C**) WMS + WP, and (**D**) RS + WP. Gray lines represent responses for each individual. Solid lines represent mean responses (95% upper and lower confidence interval). * RS + WP vs. RS at 60 min, WMS at 120 min, and WMS and WMS + WP at 180 min; *p*’s ≤ 0.045, respectively).

For the percent rate of fat oxidation, there were significant effects of time (*F*(3, 24) = 3.49, *p* = 0.031), meal type (*F*(3, 96) = 7.09, *p* < 0.001), and meal*time (*F*(9, 96) = 2.00, *p* = 0.047) (Figure 4). Bonferroni multiple comparison tests revealed RS + WP had higher fat oxidation rates compared to RS at 60 min (+23.10%, (95% CI, 6.17 to 40.03), *p* = 0.008), WMS at 120 min (+27.49%, (95% CI, 10.57 to 44.43), *p* = 0.002), and WMS and WMS + WP at 180 min (+35.76%, (95% CI, 18.83 to 52.69), *p* < 0.001 and (+17.31%, (95% CI, 0.38 to 34.24), *p* = 0.045), respectively. As with TEM there were several outliers (*n* = 5, z-scores ≥ 2.02), though their removal did not improve significance levels.

**Figure 5:**
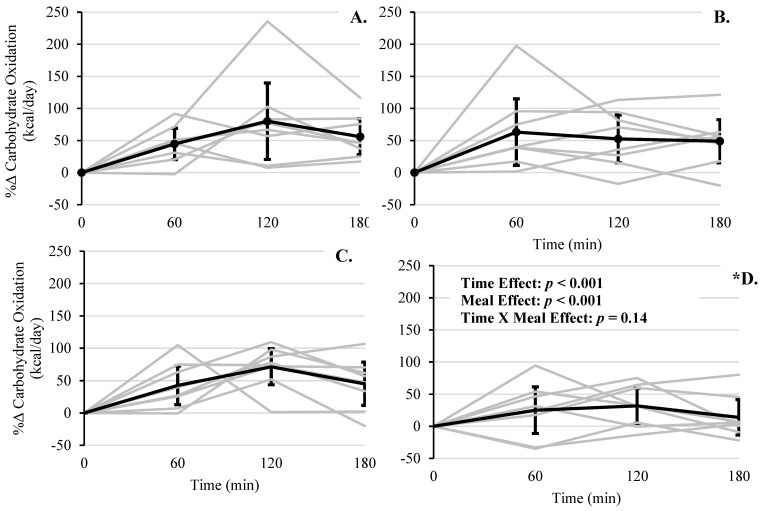
Postprandial change in percent carbohydrate oxidation across 180 min for each meal. (**A**) WMS, (**B**) RS, (**C**) WMS + WP, and (**D**) RS + WP. Gray lines represent responses for each individual. Solid lines represent mean responses (95% upper and lower confidence interval). * RS + WP% carbohydrate oxidation < WMS, RS, and WMS + WP, *p* < 0.05.

For the percent rate of carbohydrate oxidation, there was a significant main effect of time (*F*(3, 24) = 9.26, *p* < 0.001), with significant increased rates at 60 (+43.89%, (95% CI, 10.18 to 77.59%), *p* = 0.006), 120 (+58.95%, (95% CI, 25.25 to 92.65%), *p* < 0.001), and 180 min (+40.93%, (95% CI, 7.23 to 74.63), *p* = 0.011), compared to baseline (Figure 5). The main effect of meal type was also significant (*F*(3, 96) = 6.54, *p* < 0.001), with RS + WP having reduced rates compared to all three other conditions (mean differences ≥ −22.12%, *p*’s ≤ 0.01). Similar to TEM, meal × time was not significant (*F*(3, 96) = 1.54, *p* = 0.14). After removal of two outliers (i.e., z-scores ≥ 2.51) this effect remained nonsignificant.

### 3.4. Respiratory Exchange Ratio

There was a significant fixed effect of time on RER (*F*(3, 24) = 7.62, *p* = 0.001), with significant mean increases occurring at 120 (+0.05 (95% CI, 0.017 to 0.076), *p* = 0.001) and 180 (+0.04 (95% CI, 0.006 to 0.065), *p* = 0.011) minutes compared to baseline (Table 3). There was also a significant fixed effect of meal type (*F*(3, 96) = 8.13, *p* < 0.001), with RS + WP having lower overall RER values compared to the other three meals (mean differences: ≥−0.021, *p*’s ≤ 0.04). Meal × time was not significant (*F*(9, 96) = 1.08, *p* = 0.39).

### 3.5. Plasma Biomarkers

Plasma biomarkers are shown in Table 5.

**Figure 6:**
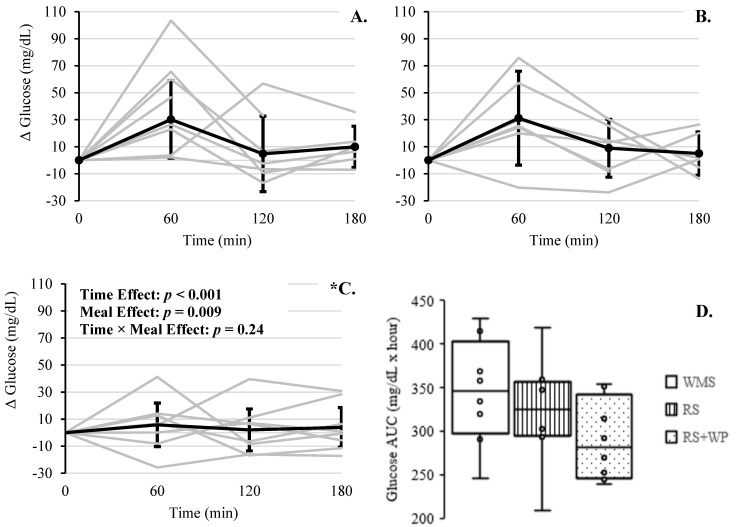
Postprandial change in glucose across 180 min for each meal. (**A**) WMS, (**B**) RS, and (**C**) RS + WP. Gray lines are responses for each individual. Solid lines are mean responses (95% upper and lower confidence interval). * WMS glucose > RS + WP, p < 0.05. (**D**). Area under the curve (AUC) over 3 h is captured for the three meals. Boxes represent interquartile range with minimum and maximum values indicated at the tips of each vertical line. The median for each meal is depicted by the horizontal line within each box. * Significance difference between AUC for WMS compared to RS + WP, *p* < 0.05.

Individual and mean changes in blood glucose, in response to each meal, are presented in Figure 6A–C. The fixed effect of time was significant (*F*(3, 83.23) = 5.92, *p* = 0.001), with significant changes in glucose concentrations occurring at 120 and 180 min compared at 60 min in the postprandial period (mean differences ≥ 19.52 mg/dL (95% CI, 5.18 to 33.94 mg/dL), *p* ≤ 0.004). The fixed effect of meal was also significant (*F*(2, 83.33) = 4.95, *p* = 0.009), with RS + WP showing reduced glucose concentrations compared to WMS (mean difference: −14.23 mg/dL (95% CI, −25.42 to −3.05 mg/dL), *p* = 0.008). Meal*time was not significant (*F*(6,83.24) = 1.35, *p* = 0.24). After removal of two outliers (i.e., z-scores ≥ 2.45), this interaction effect remained nonsignificant. Blood glucose AUC differed significantly between the three reported study meals (*F*(2, 16) = 4.21, *p* = 0.034; Figure 6D), with RS + WP having significantly lower glucose AUC compared to WMS (mean difference: −16.02%, absolute −55.27 mg/dL, (95% CI, −106.40 to −4.13 mg/dL), *p* = 0.032).

**Figure 7:**
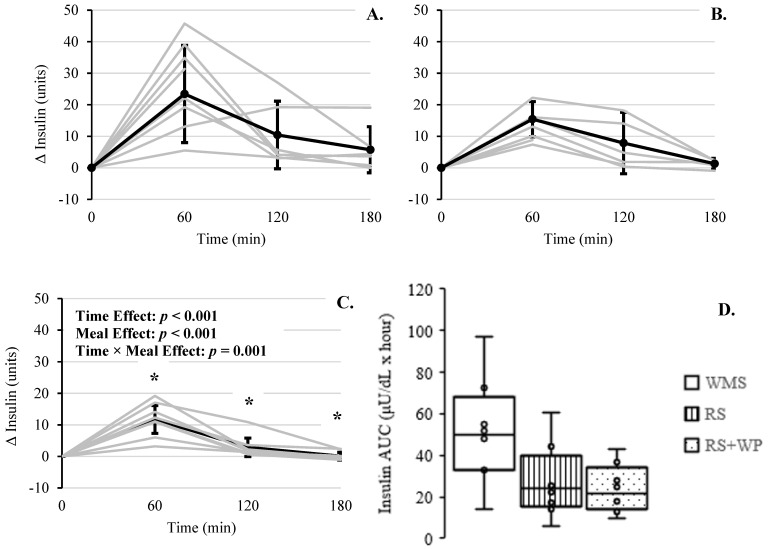
Postprandial change in plasma insulin across 180 min for each meal. (**A**) WMS, (**B**) RS, and (**C**) RS + WP. Gray lines represent responses for each individual. Solid lines represent mean responses (95% upper and lower confidence interval). * Significant difference compared to WMS, *p* < 0.05. (**D**) Area under the curve (AUC) over 3 h is shown for the three meals. Boxes represent the interquartile range for each meal, and minimum and maximum values are indicated at the tips of each vertical line. The median for each meal is depicted by the horizontal line within each box. * Significant difference compared to RS and RS + WP, *p* < 0.05.

Individual and mean changes in plasma insulin over time, in response to each meal, are presented in Figure 7A–C. The effects of time (*F*(3, 23.96) = 34.83, *p* < 0.001), meal type (*F*(3, 64.14) = 11.69, *p* < 0.001), and meal × time (*F*(6, 60.89) = 4.34, *p* = 0.001) were all significant. Bonferroni multiple comparison tests revealed RS + WP had lower mean insulin levels compared to WMS at 60 min (−14.63, (95% CI, −19.12 to −10.12), *p* < 0.001), 120 min (−6.59, (95% CI, −11.28 to −1.91), *p* = 0.007), and 180 min (−4.27, (95% CI, −9.38 to −0.013), *p* = 0.049). In addition, the mean 3 h plasma insulin AUC differed significantly (F(2, 16) = 12.82, *p* < 0.001; Figure 7D), with both RS + WP and RS having significantly lower insulin AUC compared to WMS (mean differences: −53.05%, absolute −26.83 units, (95% CI, −42.24 to −11.42 units), *p* = 0.001, and −46.19%, absolute −23.36 units, (95% CI −38.76 to −7.95 units), *p* = 0.003, respectively).

GIP, GRL, and PYY all showed significance for the effect of time (*p*’s < 0.001); however, there were no significant effects of meal and meal × time for GIP, GRL, and PYY (*p*’s ≥ 0.08 and *p*’s ≥ 0.60, respectively; Table S1). In contrast, there was a significant effect of meal on GLP-1 (*F*(2, 58.49) = 5.29, *p* = 0.008), with RS + WP showing significantly lower plasma GLP-1 levels compared to RS (mean difference: −0.84 pM (95% CI, −5.01 to −0.68 pM), *p* = 0.006). However, there was no significant fixed effect of meal × time (*F*(6, 58.70) = 0.47, *p* = 0.83).

### 3.6. Feelings of Hunger, Satiation and Desire to Eat

All satiation and hunger ratings significantly decreased (main effects of time: *F*(3, 24) = 6.92 to 12.12, *p*’s ≤ 0.002) at 60 and 120 min following consumption of each pancake meal test (Table S1). However, the fixed effects of meal and meal × time were not significant (*F*(3, 96) = 0.14 to 2.49, *p*’s ≥ 0.06 and *F*(9, 96) = 0.63 to 0.95, *p*’s ≥ 0.49, respectively). The feeling of fullness significantly increased over time (fixed effect of time, F(3, 24) = 11.02, *p* < 0.001) at 60 and 120 min following meal ingestion, while the fixed effects of meal and meal × time were not significant (*F*(3, 96) = 1.96, *p* = 0.13 and *F*(3, 96) = 0.98, *p* = 0.47, respectively).

## 4. Discussion

The major aim of the current study was to quantify the effects of pancake meals containing non-RS constituents or RS4 meals with and without greater protein contents on postprandial thermogenesis (TEM), fuel utilization, satiety, and gastro-entero-pancreatic hormones in healthy middle-aged men. The main findings reveal that RS + WP, compared to other test meals, elicited (1) a larger thermogenic effect; (2) greater rate of fat oxidation and lower CHO oxidation; and (3) reduced postprandial circulating glucose and insulin levels. Subjective feelings of hunger, satiation, and desire to eat were not different among the four meals. Taken together, these findings show acute ingestion of a solid food meal containing RS + WP elicits a significantly greater thermogenic response (total calorie burn), fat oxidation rates, and lower glucose/insulin responses compared to whole food meals of equal caloric and macronutrient composition. These results may have important implications regarding the incorporation of functional ingredients including resistant starches and isolated dairy proteins into various food matrices for weight control and mitigating metabolic-related health conditions (e.g., diabetes, obesity, inflammation).

The major finding of the current study is the significant magnitude of increase (>76%) in TEM with RS + WP compared to the other isocaloric test meal conditions. This clearly reflects a metabolic advantage of combined resistant starch with whey protein meals. The likely mechanism for this heightened thermic response is the different digestion and absorption and subsequent short-chain fatty acid (SCFA) production of RS. Specifically, the total macronutrient amount and proportion of calories entering circulation may be responsible for the drastic increase in thermogenesis with RS compared with isocaloric non-RS containing meals. Thus, further research needs to elucidate whether the greater TEM response with RS meals is due to altered gut metabolism or simply a reflection of altered amounts of calories/macronutrients being metabolized, or a combination of both. Further, given the addition of WP was necessary for this greater TEM response, there seems to be a supra-additive effect of both together compared to each alone. Our lab has previously demonstrated the “metabolic advantage” of increased TEM from consuming protein pacing meals/snacks compared to comparable isocaloric non-protein pacing meals [4]. In addition, data from our laboratory have previously reported increased fat oxidation, PYY, and increased satiety and fullness following consumption of combined dietary resistant starch and whey protein in lean and overweight/obese females [6]. Using identical study methodology as the current investigation, in females we did not find the same effect of significantly increased post-prandial energy expenditure nor the reductions in postprandial glucose and insulin concentrations. Though in contrast to the current study, RS + WP did increase the feelings of fullness, while decreasing the feeling of hunger versus non-whey conditions. The divergent findings between males and females highlight potential sex differences that may warrant further investigation. Interestingly, no previous rationale for sex differences in these responses following consumption of identical test meals exists. Thus, conflicting findings remain unclear at this time. In sum, the combination of RS and WP may elicit enhanced gut (SCFA production) and macronutrient (calorie) metabolism that results in greater postprandial thermogenic response compared to isocaloric meal challenges and thus may serve as a highly effective, long-term nutrition strategy to aid with weight control, body composition, and cardiometabolic health outcomes.

Our results align with previous research utilizing RS in a solid food matrix meal challenge, particularly in reducing postprandial elevations in blood glucose and/or plasma insulin compared to matched carbohydrate controls [19,20,27,28,29,30]. Similar in research design to our study, Shimotoyodome et al. (2011) provided participants with a mixed meal pancake breakfast challenge containing a comparable amount of RS (38 g) or WMS (38 g) [19]. Not only did the RS meal elicit significantly lower postprandial glucose and insulin concentrations, but postprandial thermogenesis and fat utilization were also significantly elevated in healthy male participants. More recently in a double-blind, randomized crossover study, Mah et al. (2018) reported RS4, as a constituent of a breakfast bar, reduced glucose and insulin AUC [28]. Findings from these studies, as well as the present study, have important clinical relevance due to the strong relationship between poorly controlled postprandial blood glucose control and development of diabetes. Indeed, more recent data point to a strong correlation between poor postprandial blood glucose control and the presence of coronary heart disease [31].

The current findings support the replacement of a certain amount of available carbohydrate with RS4 to help lower postprandial glucose, as this may reduce the amount of carbohydrate contributing to blood glucose. This assertion is supported by animal studies that have shown decreased gastrointestinal transit time of RS-containing carbohydrates [32], which in turn likely allows portions of the available carbohydrate to escape digestion and absorption in the small intestine. Once in the lower portions of the gastrointestinal tract, RS may be used as substrate for microbial fermentation and production of SCFAs, as well as other metabolites with beneficial metabolic properties. Indeed, supplementing with RS4 over a 12-week period in participants with signs of metabolic symptoms significantly altered the composition of the gut microbiome, including the enrichment of bacteria with starch-degrading enzymes and increased fecal SCFAs (such as butyrate, propionate, valerate, isovalerate, and hexanoate) [33]. In addition, cholesterol, glycemic (fasting glucose, glycosylated hemoglobin) and proinflammatory markers, along with as anthropometric measures (waist circumference, percent body fat) were also reduced in the RS4 group versus the control group post intervention. These positive outcomes have been hypothesized to be, in part, mediated by SCFAs via microbial production from RS fermentation in the gut [34]. These changes may also promote anti-inflammatory and signaling activity, both locally in the gut and systemically, which warrants further investigation in future studies.

Shimotoyodome et al. (2011) reported reduced postprandial GIP levels in the RS group, which may mediate some of the beneficial effects of RS [19], and this is supported by others showing dietary carbohydrates and fats stimulate postprandial increases in GIP levels [35,36,37]. GIP has been proposed to stimulate efficient fat deposition [38,39], whereas inhibition of GIP signaling increases fat oxidation and energy expenditure, as well as reduces high-fat diet-induced obesity in mice [40,41]. Daousi et al. (2009) demonstrated elevated blood GIP levels are associated with lower resting energy expenditure (REE) in healthy humans [42]. Interestingly, our data did not show a difference between meal conditions on GIP. However, we did note RS + WP had a significantly lower GLP-1 compared to RS. As an anorexigenic gastrointestinal peptide, the lower GLP-1 levels in response to the RS + WP meal is surprising given whey protein has been well characterized to stimulate GLP-1 release in acute meal challenges over carbohydrate controls [43,44,45]. However, in our previous work using the same research methodology and test meals we did not find a significant difference between conditions in females [6].

In contrast to our test meals, the test meals provided by Shimotoyodome et al. (2011) and Mah et al. (2018) contained lower comparative amounts of protein [19,28]. As a functional food component used in meal replacement products and other food applications for the promotion of weight loss/maintenance, whey protein has been well-described to have positive effects on both body composition and cardiometabolic outcomes [4,5,22,46]. Currently, there is a paucity of research that has examined acute metabolic outcomes from the combination of both RS and isolated dairy proteins as functional ingredients in various processed food applications.

Several limitations in the present study should be noted. First, the number of participants was *n* = 8, and only middle-aged men were included, which limits the applicability of our findings to women and other age ranges. We noted several outliers (i.e., z-scores ≥ 2.0) whose removal improved the trend toward significance for the interaction effects of postprandial change in TEM. While important to note, these outliers where included in our formal analysis. Such findings are not uncommon in small sample sizes and highlight the interindividual variability in metabolic response to these meal challenges and importance of displaying these data visually (i.e., individual response curves in Figure 2, Figure 4, Figure 5, Figure 6 and Figure 7). Second, both the resistant starch and whey protein were supplemental powders; therefore, the physiological effects, including energy expenditure, hormone levels, and satiety, of powder may differ from whole food sources. Third, the glycemic load and index of the test meals were not calculated; thus, the contribution of these factors to the major outcomes of TEM, fuel utilization, hormone, and hunger responses is missing. Fourth, as mentioned above in the methods, plasma variables for WMS + WP were not obtained; therefore, the independent effects of whey protein on satiety/hunger hormones were not established. Lastly, subjective feelings of hunger/satiation did not differ significantly among conditions, which may limit the long-term efficacy of WMS + WP to sufficiently support a caloric deficit and promote weight loss.

## 5. Conclusions

Overall, the current study highlights the benefit of certain combinations of functional foods on postprandial thermogenesis and plasma glucose and insulin responses. Specifically, an acute meal challenge containing both RS and WP favorably increases postprandial thermogenesis and fat oxidation compared to an isocaloric WMS, WMS + WP, and RS meal. Moreover, the RS + WP pancake meal resulted in lower insulin responses than the other meals. The extent of increased energy expenditure (>20 kcals/180 min) and fat oxidation (>30%) of RS + WP, compared to the other isocaloric meals, suggests this response may be biologically relevant and serve as a critical strategy to prevent fat deposition over the long term by reducing total fat balance during habitual meal intake environments. The results support replacing refined starch with a “functional” form like RS4, along with WP, in an otherwise “processed” food matrix may promote favorable metabolic effects to enhance weight control and body composition. It is prudent to confirm similar findings in obese subjects using studies designed to explore precise mechanisms of action.

## Figures and Tables

**Table 1:** Pancake test meal nutritional analysis.

	WMS (Control)	WMS + WP	RS	RS + WP
Energy (kcal)	397.0	397.0	397.0	397.0
Waxy maize starch (g)	45.0	45.0	–	–
Resistant starch ^a^ from waxy maize starch (g)	–	–	40.0	40.0
Whey protein (g)	–	20.5	–	20.5
Sucrose (g)	4.5	4.5	4.5	4.5
Maltodextrin (g)	10.7	10.7	10.7	10.7
Milk powder (g)	21.1	0.90	21.1	0.90
Egg (g)	50.0	50.0	50.0	50.0
Baking powder (g)	4.8	4.8	4.8	4.8
Total carbohydrate (g)	73.0	60.0	73.0	60.0
Total fat (g)	5.0	5.5	5.0	5.5
Total protein (g)	15.0	26.8	15.0	26.8
Total fiber (g)	0.0	0.0	0.0	0.0

^a^ In the form of hydroxypropyl-distarch phosphate.

**Table 2:** Subject characteristics ^a^.

*N*	8
Age (years)	49.00 ± 13.61
Weight (kg)	91.34 ± 11.77
Height (cm)	180.49 ± 5.83
BMI	28.16 ± 5.071
Percent fat mass (%)	26.85 ± 10.17
Systolic BP (mmHg)	123.25 ± 8.65
Diastolic BP (mmHg)	76.50 ± 4.75
Resting HR (bpm)	58.75 ± 8.12
Glucose (mg/dL)	91.75 ± 10.38
Total Cholesterol (mg/dL)	181.50 ± 27.88
HDL (mg/dL)	44.75 ± 7.78
LDL (mg/dL)	114.50 ± 29.41
Triglycerides (mg/dL)	109.63 ± 55.67
TC/HDL ratio	4.12 ± 0.87

^a^ Data presented as mean ± standard deviation.

**Table 3:** Effects of meal type on mean values of metabolic outcome measurements at baseline and 60, 120, and 180 min in the postprandial period ^a^.

Outcome Variable	Meal	Baseline		60 min		120 min		180 min	
		Mean	95% CI	Mean	95% CI	Mean	95% CI	Mean	95% CI
RMR and TEM (kcal/minute) ^b^	WMS	1.17	(1.05–1.29)	1.33	(1.21–1.45)	1.31	(1.19–1.43)	1.23	(1.11–1.35)
	RS	1.17	(1.05–1.29)	1.38	(1.26–1.49)	1.29	(1.17–1.41)	1.29	(1.18–1.42)
	WMS + WP	1.15	(1.03–1.27)	1.34	(1.22–1.46)	1.28	(1.16–1.40)	1.26	(1.14–1.38)
	RS + WP *	1.25	(1.13–1.37)	1.42	(1.29–1.54)	1.41	(1.29–1.53)	1.34	(1.22–1.46)
RER	WMS	0.85	(0.83–0.88)	0.86	(0.83–0.89)	0.9	(0.87–0.93)	0.89	(0.86–0.92)
	RS	0.81	(0.79–0.84)	0.88	(0.85–0.90)	0.87	(0.85–0.90)	0.87	(0.84–0.89)
	WMS + WP	0.83	(0.80–0.86)	0.86	(0.83–0.88)	0.88	(0.85–0.91)	0.87	(0.84–0.89)
	RS + WP *	0.82	(0.79–0.85)	0.84	(0.81–0.87)	0.85	(0.82–0.88)	0.86	(0.81–0.87)
Carbohydrate Oxidation Rate (kcal/day)	WMS	892	(660–1124)	1076	(844–1308)	1282	(1050–1514)	1135	(903–1367)
	RS	632	(400–864)	1227	(995–1458)	1103	(871–1335)	1061	(829–1293)
	WMS + WP	821	(589–1053)	1110	(878–1342)	1273	(1041–1505)	1095	(863–1327)
	RS + WP *	664	(432–896)	941	(709–1173)	957	(725–1189)	839	(607–1071)
Fat Oxidation Rate (kcal/day)	WMS	806	(636–977)	874	(704–1045)	615	(445–786)	640	(470–811)
	RS	1057	(886–1227)	767	(596–937)	756	(585–926)	880	(710–1051)
	WMS + WP	982	(812–1153)	934	(763–1105)	768	(597–938)	810	(640–981)
	RS + WP	1003	(832–1173)	993 **	(822–1163)	895 **	(724–1065)	982 **	(811–1153)

^a^ Effects based on estimated marginal means. Bonferroni adjustments were conducted for multiple comparisons. ^b^ RMR: Resting metabolic rate, taken at baseline. TEM: Thermic effect of meal, taken at 60, 120, and 180 min postprandially. * Significant main effect of meal, vs. WMS, RS, WMS + WP, all *p*’s < 0.05. ** Significant differences: at 60 min vs. RS, *p* = 0.008; at 120 min vs. WMS, *p* = 0.002; at 180 min vs. WMS and WMS + WP, *p*’s ≤ 0.045. WMS: Waxy maize starch; RS: Resistant starch; WMS + WP: Waxy maize starch + whey protein; RS + WP: Resistant starch + whey protein; RER: Respiratory exchange ratio.

**Table 4:** Calories consumed and postprandial response (kcals burned/180 min) during thermic effect of the four test meals (TEM) ^a^.

Meal	Kcal Consumed	RMR ^b^	60 min iAUC TEM ^c^	120 min iAUC TEM	180 min iAUC TEM	Total AUC TEM ^d^
WMS	397	1686 (1490–1881)	4.39 (−0.29–9.08)	12.53 (7.84–17.21)	8.70 (4.02–13.39)	25.61 (14.31–36.91)
RS	397	1682 (1507–1858)	5.74 (1.05–10.42)	14.48 (9.79–19.16)	9.23 (4.54–13.91)	29.44 (18.14–40.74)
WMS + WP	397	1659 (1447–1871)	4.54 (−0.15–9.22)	12.00 (7.32–6.69)	8.10 (3.42–12.79)	24.64 (13.34–35.94)
RS + WP *	397	1692 (1513–1870)	6.83 (2.14–11.51)	20.36 (15.68–25.05)	17.93 (13.24–22.61)	45.11 (33.81–56.41) *

^a^ Effects based on estimated marginal means, actual values displayed (mean (95% lower-upper confidence intervals)). ^b^ RMR: Resting metabolic rate taken at baseline, kcals/day. ^c^ iAUC TEM: Incremental area under the curve for the thermic effect of meal for 0–60, 60–120, and 120–180-min periods, postprandially, kcals/minute. ^d^ Total area under the curve calculated for the 0–180-min period, total TEM kcals. WMS: Waxy maize starch; RS: Resistant starch; WMS +WP: Waxy maize starch + whey protein; RS + WP: Resistant starch + whey protein. * Significant main effect of meal; RS + WP > WMS, WMS + WP; *p* < 0.05.

**Table 5:** Effects of meal type on mean values of plasma biomarkers baseline and 60, 120, and 180 min in the postprandial period ^a^.

Outcome Variable	Meal	Baseline		60 min		120 min		180 min	
		Mean	95% CI	Mean	95% CI	Mean	95% CI	Mean	95% CI
Glucose (mg/dL)	WMS	110.41	(94.68–126.13)	133.11	(117.39–148.84)	99.95	(83.49–116.40)	101.42	(84.96–117.87)
	RS	105.06	(89.34–120.79)	121.64	(105.18–138.09)	99.87	(84.15–115.59)	96.65	(79.26–114.05)
	RS + WP *	100.86	(85.14–116.89)	97.57	(81.14–116.59)	93.83	(78.10–109.55)	95.7	(79.98–111.43)
Insulin (units)	WMS	3.52	(−1.19–8.23)	29.43	(24.72–34.14)	12.53	(7.64–17.41)	7.83	(2.95–12.72)
	RS	2.87	(−1.84–7.58)	16.89	(12.01–21.78)	8.52	(3.64–13.41)	3.51	(–1.59–8.63)
	RS + WP *	2.86	(−1.85–7.57)	14.80 **	(10.09–19.51)	5.94 **	(1.23–10.65)	3.14 **	(–1.57–7.85)
GIP (pmol/L)	WMS	35.26	(0.50–70.01)	184.98	(150.23–219.73)	148.32	(112.57–184.06)	89.31	(54.56–124.06)
	RS	36.36	(1.61–71.11)	169.97	(134.23–205.71)	131.84	(97.08–166.59)	80.66	(43.62–117.69)
	RS + WP	26.69	(−8.06–61.45)	157.42	(122.66–192.17)	133.00	(98.25–167.76)	74.78	(40.03–109.54)
GLP-1 (pM)	WMS	6.63	(3.03–10.22)	11.71	(7.85–15.77)	11.32	(7.62–15.03)	9.93	(6.22–13.63)
	RS	7.44	(3.85–11.03)	13.51	(9.80–17.22)	15.08	(11.22–18.93)	10.73	(6.87–14.59)
	RS + WP	6.13	(2.53–9.72)	10.13	(6.53–13.72)	10.25	(6.66–13.84)	8.88	(5.28–12.47)
GRL (pM)	WMS	375.47	(253.34–497.59)	241.84	(118.37–365.31)	321.06	(197.62–444.49)	377.53	(255.41–499.66)
	RS	413.76	(291.64–535.89)	278.22	(154.77–401.66)	367.44	(245.31–489.56)	411.33	(286.09–536.57)
	RS + WP	400.08	(277.96–522.20)	289.58	(166.11–413.05)	330.52	(208.39–452.64)	426.69	(304.57–548.81)
PYY (pg/mL)	WMS	32.63	(14.25–51.02)	45.16	(26.57–63.76)	41.87	(23.49–60.26)	44.38	(26.01–62.77)
	RS	27.86	(9.62–46.08)	40.78	(22.39–59.16)	44.48	(26.76–64.56)	45.66	(26.76–64.56)
	RS + WP	29.36	(11.13–47.59)	34.11	(15.88–52.34)	36.36	(18.13–54.59)	43.34	(24.96–61.72)

^a^ Effects based on estimated marginal means. Bonferroni adjustments were conducted for multiple comparisons. * Significant effect of meal, vs. WMS *p* < 0.05. ** Significant interaction effect, vs. WMS *p* < 0.05. WMS: Waxy maize starch; RS: Resistant starch; RS + WP: Resistant starch + whey protein; GIP: Gastric inhibitory peptide; GLP-1: Glucagon-like polypeptide-1; GRL: Ghrelin; PYY: Peptide YY.

## Data Availability

The data presented in this study are available on request from the corresponding author. The data are not publicly available due to privacy restrictions.

## Supplementary Materials

The following are available online at https://www.mdpi.com/2304-8158/10/3/537/s1. Table S1: Hunger and satiety ratings, Effects of meal type on mean values of hunger and satiety rating outcome measurements at baseline and 60, 120, and 180 minutes in the postprandial period.

## Abbreviations

The following abbreviations are used in this manuscript:

AUC	area under the curve
BMI	body mass index
GIP	gastric inhibitory peptide
GLP-1	glucagon-like polypeptide-1
GRL	ghrelin
LEP	leptin
PYY	peptide YY
RER	respiratory exchange ratio
RMR	resting metabolic rate
RS4	resistance starch, type 4
RS	resistant starch, type 4
RS + WP	resistant starch and whey protein
SCFA	short-chain fatty acid
TEM	thermic effect of a meal
VAS	visual analog scale
WMS	waxy maize starch
WMS + WP	waxy maize starch and whey protein
