# Supplementary material for: Resistant Starch Combined with Whey Protein Increases Postprandial Metabolism and Lowers Glucose and Insulin Responses in Healthy Adult Men
Source: Foods. 2021 Mar 5;10(3):537. doi: 10.3390/foods10030537 (PMC8000721; doi:10.3390/foods10030537)
Supplement: Supplementary file 1 [file foods-10-00537-s001.pdf]

Table S1. Effects of meal type on mean values of hunger and satiety rating outcome measurements at baseline and 60, 120, and 180 minutes in the postprandial period.[a]

		Baseline		60 min		120 min		180 min	
**Outcome Variable**	**Meal**	**Mean**	**95% CI**	**Mean**	**95% CI**	**Mean**	**95% CI**	**Mean**	**95% CI**
Hunger (mm)	WMS	59.13	(46.66-71.59)	36.86	(24.41-49.34)	38.38	(25.91-50.84)	39.25	(26.78-51.72)
	RS	48.00	(35.53-60.47)	26.16	(13.66-38.59)	32.63	(20.16-45.09)	39.63	(27.46-52.09)
	WMS + WP	48.25	(35.78-60.72)	29.38	(16.91-41.84)	38.00	(25.53-50.47)	42.38	(29.91-54.84)
	RS + WP	42.88	(30.41-55.34)	27.75	(15.28-40.22)	36.50	(24.03-48.97)	42.50	(30.03-54.97)
Desire to Eat (mm)	WMS	60.13	(46.33-73.92)	38.00	(24.20-51.79)	37.50	(23.70-51.29)	47.00	(33.20-60.79)
	RS	51.00	(37.20-64.79)	25.37	(11.58-39.17)	37.88	(24.08-51.67)	39.38	(25.58-53.17)
	WMS + WP	47.44	(33.64-61.24)	25.81	(12.01-39.61)	38.63	(24.83-52.42)	47.69	(33.89-61.48)
	RS + WP	51.63	(37.83-65.42)	35.25	(21.45-49.05)	37.86	(24.08-51.67)	44.12	(30.33-57.92)
Quantity Food Think Can Eat (mm)	WMS	64.75	(54.75-74.75)	45.50	(35.49-55.50)	45.38	(35.37-55.38)	47.75	(37.75-57.75)
	RS	62.63	(52.62-72.63)	37.50	(27.49-47.50)	48.87	(38.87-58.88)	54.75	(44.75-64.75)
	WMS + WP	64.25	(54.25-74.25)	40.63	(30.62-50.63)	46.63	(36.62-56.63)	52.00	(41.99-62.00)
	RS + WP	55.50	(45.49-65.50)	39.56	(29.56-49.57)	47.50	(37.49-57.50)	55.13	(45.12-65.13)
Feeling of Fullness (mm)	WMS	29.25	(18.51-39.99)	44.00	(33.26-54.74)	47.12	(36.39-57.86)	41.37	(30.64-52.11)
	RS	32.00	(21.26-42.74)	54.87	(44.14-65.61)	47.13	(36.39-57.86)	39.75	(29.01-50.45)
	WMS + WP	33.56	(22.82-44.30)	46.75	(36.01-57.49)	37.25	(26.51-47.99)	29.63	(18.89-40.36)
	RS + WP	33.75	(23.01-44.48)	56.25	(45.51-66.99)	41.25	(30.51-51.99)	42.00	(31.26-52.74)

[a]Effects based on estimated marginal means. Bonferroni adjustments were conducted for multiple comparisons.
WMS: Waxy maize starch; RS: Resistant starch; WMS + WP: Waxy maize starch + whey protein; RS + WP: Resistant starch + whey protein.
